# Research Progress on the Inflammatory Effects of Long Non-coding RNA in Traumatic Brain Injury

**DOI:** 10.3389/fnmol.2022.835012

**Published:** 2022-03-10

**Authors:** Jian-peng Wang, Chong Li, Wen-cong Ding, Gang Peng, Ge-lei Xiao, Rui Chen, Quan Cheng

**Affiliations:** ^1^Department of Neurosurgery, The Affiliated Nanhua Hospital, Hengyang Medical School, University of South China, Hengyang, China; ^2^Department of Neurosurgery, Xiangya Hospital, Central South University, Changsha, China; ^3^Department of Clinical Pharmacology, Xiangya Hospital, Central South University, Changsha, China; ^4^National Clinical Research Center for Geriatric Disorders, Xiangya Hospital, Central South University, Changsha, China

**Keywords:** traumatic brain injury, long non-coding RNA, inflammatory pathways, immune response, therapy

## Abstract

Globally, traumatic brain injury (TBI) is an acute clinical event and an important cause of death and long-term disability. However, the underlying mechanism of the pathophysiological has not been fully elucidated and the lack of effective treatment a huge burden to individuals, families, and society. Several studies have shown that long non-coding RNAs (lncRNAs) might play a crucial role in TBI; they are abundant in the central nervous system (CNS) and participate in a variety of pathophysiological processes, including oxidative stress, inflammation, apoptosis, blood-brain barrier protection, angiogenesis, and neurogenesis. Some lncRNAs modulate multiple therapeutic targets after TBI, including inflammation, thus, these lncRNAs have tremendous therapeutic potential for TBI, as they are promising biomarkers for TBI diagnosis, treatment, and prognosis prediction. This review discusses the differential expression of different lncRNAs in brain tissue during TBI, which is likely related to the physiological and pathological processes involved in TBI. These findings may provide new targets for further scientific research on the molecular mechanisms of TBI and potential therapeutic interventions.

## Introduction

Traumatic Brain Injury (TBI) is the direct or indirect effect of external force on the head, causing mechanical damage to the brain tissue, and a series of secondary pathological injuries. It can have different clinical manifestations, such as changes in brain morphology, limb dysfunction (Jang and Seo, 2020), endocrine changes (Li and Sirko, 2018), abnormal blood coagulation (Albert et al., 2019), mental disorders (Pavlovic et al., 2019), and changes in consciousness. Its disability rate and death rate are the highest among limb injuries, costing hundreds of billions of dollars each year (Rubiano et al., 2015). Thus, this public health problem must be urgently solved. However, to the mechanism of secondary brain injury is complex, and remains unclear. All clinical treatment measures are aimed at reducing secondary brain injury, but their efficacy is limited.

Generally, long non-coding RNAs (lncRNAs) are non-coding RNA (ncRNA) transcripts greater than 200 nucleotides (nt) in length. These non-coding sequences were identified in the 1970s (Iyer et al., 2015; Ren et al., 2020). At present, the general understanding of evolutionary biology states that lncRNAs have no biological function and are, therefore, “junk DNA” (Chen et al., 2019). However, several studies have shown that this “junk DNA” has important biological functions. The expression of lncRNAs in the injured brain is different from that in healthy tissue, and this plays an important role in secondary injury. This has aroused the interest of scholars in the role of lncRNAs in TBI. Here, we review the inflammatory effects mediated by different lncRNAs in TBI, and discuss the mechanisms of secondary brain injury (Shao et al., 2019). Targeted lncRNA regulation or intervention of target downstream molecules are expected to improve TBI treatment and open new paths for drug development.

## Mechanism of Traumatic Brain Injury

The clinical manifestation of TBI, which is caused by direct or indirect mechanical trauma to the head, varies depending on the mechanism and severity of the injury (Gaddam et al., 2015; Hiebert et al., 2015). Patients with TBI exhibit the following two types of injuries: primary injury resulting from direct traumatic damage to the brain tissue and including concussion, cerebral contusion, intracranial hematoma, and axonal injury; secondary brain injury resulting from damage to the brain microstructure following a series of pathological processes accompanying the primary brain injury, including inflammatory factor release, oxidative stress, calcium overload, impairment of mitochondrial function, ferroptosis, apoptosis, pyroptosis, and autophagy (Kaur and Sharma, 2018; Jha et al., 2019) and promoting neuronal death (Dixon, 2017).

## Epidemiology of Traumatic Brain Injury

Statistics show that more than 50 million people worldwide experience TBI every year, and about half of the global population is likely to suffer one or more TBI in their lifetime (Maas et al., 2017). TBI causes approximately 400 billion U.S. dollars in losses to the global economy every year, accounting for 0.5% of the world’s GDP (Taylor et al., 2017). The incidence of TBI in Central Europe, Eastern Europe, and Central Asia is significantly higher than that in other parts of the world. Syria is the country with the highest age-standardized incidence of TBI, followed by Slovenia and the Czech Republic (GBD 2016 Traumatic Brain Injury and Spinal Cord Injury Collaborators, 2019b), the annual incidence of TBI in Europe is 47.3/100,000–849/100,000, and the case fatality rate is 3.3/100,000–28.1/100,000 (Brazinova et al., 2021). China is the most populous country worldwide, according to the National Bureau of Statistics of China, the total population of China exceeded 1.390 billion, which accounted for approximately 18% of the global population, in 2017 (National Bureau of Statistics of People’s Republic of China, 2018). The incidence of TBI in China is higher than that in most other countries (Maas et al., 2017). The population-based mortality rate of TBI in China is approximately 1.3/10,000 with traffic accidents contributing to the majority of the cases (Gao et al., 2020) followed by contributions from falls, violence, and other causes (Jiang et al., 2019). The poor survival and prognosis of patients with severe central nervous system (CNS) injury can be attributed to the complex and diverse pathological mechanisms in different degrees of brain injury involving multiple organs and systems, limited conservative and surgical treatment modalities, the poor therapeutic efficacy of traditional treatment (Campos-Pires et al., 2015) and expensive medical costs. Thus, TBI is a major burden to patients and their families, and society. Therefore, there is a need to develop novel and effective therapeutic strategies to increase the patient survival rate, improve prognosis, and mitigate the burden on families and society.

## Long Non-Coding RNAs

The Human Genome Project revealed that the human genome comprises more than three billion nucleotide sequences, with approximately 2% of the transcripts encoding proteins. Approximately 98% of the transcripts that do not encode proteins are called non-coding RNAs (ncRNAs) (ENCODE Project Consortium, 2012; Huang X. et al., 2018); ncRNAs can be classified into short-stranded ncRNAs (short/small ncRNAs; length < 200 nt) [such as small nuclear RNA, small nucleolar RNAs (snoRNAs), microRNAs (miRNAs), PIWI-interacting RNAs (piRNAs), small interfering RNA (siRNAs)] and long-stranded ncRNAs (>200 nt). However, this classification is not accurate because lncRNAs <200 nt have been identified. Recent studies have demonstrated that some special lncRNAs contain short open reading frames that can encode some small proteins (Chen, 2016). The ncRNA whose length is greater than 200 nt is called lncRNA. The lncRNA transcribed by RNA polymerase II, has a conserved secondary structure (Fatica and Bozzoni, 2014). Additionally, lncRNAs have specific primary and tertiary structures and are involved in various biological processes, including gene expression and regulation.

### Classification of Long Non-coding RNAs

Researchers have classified lncRNAs to understand their properties (St Laurent et al., 2015).

First, ncRNAs can also be classified according to their functions as follows: (a) Constitutive or housekeeping ncRNAs [ribosomal RNA (rRNA) and transfer RNA (tRNA), and small cytoplasmic RNA]; (b) Regulatory ncRNAs [long-stranded ncRNAs (such as lncRNAs) and short-stranded ncRNAs (siRNAs, miRNA, and piRNA)].

Second, lncRNAs can be classified according to their positions relative to the protein-coding regions and messenger motifs, and their correlation with gene enhancer regulatory elements: intergenic lncRNAs, intronic lncRNA, sense lncRNA, antisense lncRNAs, and bidirectional lncRNAs (Ponting et al., 2009; Ma et al., 2013; Wang and Li, 2013).

The development of high-throughput molecular technologies, such as probe microarray (Lee and Kikyo, 2012), has increased our understanding of the lncRNA-regulated genes. The modulation of the lncRNA-regulated related genes and pathways can aid in the development of novel clinical treatment modalities.

### Structure of Long Non-coding RNAs

Researchers are only now beginning to understand the diverse regulatory roles played by lncRNAs, which are distributed across the eukaryotes, such as animals, plants, yeast (Houseley et al., 2008; Swiezewski et al., 2009; Sendler et al., 2013; Shi et al., 2013), and viruses (Ma et al., 2013). Similar to other RNAs, lncRNAs exhibit a primary structure, which is determined by the nucleotide sequence and the complementary base pairing principle. The primary structure is critical for the binding of lncRNAs to the target genes to regulate their transcription or translation (Qian et al., 2019). Similar to mRNAs, most lncRNAs contain cap structures, spliceosomes, and polyadenylate tails (Mattick and Rinn, 2015). Enhancer RNAs (eRNAs) comprise a cap structure but not the spliceosome or polyadenylate tail (Miladi et al., 2019). The secondary structure of lncRNAs (also called stem-loop structure) involves a double-stranded stem formed between paired bases and an unpaired single-stranded loop. LncRNAs exhibit several secondary structures, such as the stem, hairpin loop, convex loop, inner loop, and multi-branched loop, depending on their nucleotide composition (Johnsson et al., 2014; Zampetaki et al., 2018; Singh et al., 2019). The functions of lncRNAs are dependent on their secondary structure and tertiary structure (spatial structure) (Novikova et al., 2012a). Limited studies have reported the tertiary structures of lncRNAs, which can be attributed to vast numbers, large molecular weights, poor *in vitro* stability, and difficulty in crystallization of lncRNAs. The understanding of the advanced structure of lncRNAs is often based on the correlation studies of the well-studied lncRNAs: HOX transcript antisense RNA (HOTAIR) and metastasis-associated lung adenocarcinoma transcript 1(MALAT1) (Novikova et al., 2012b; Novikova et al., 2013; Sun et al., 2018). An in-depth structural analysis will provide useful insights into the function and mechanism of lncRNAs.

### Functions of Long Non-coding RNAs

**FIGURE 1:**
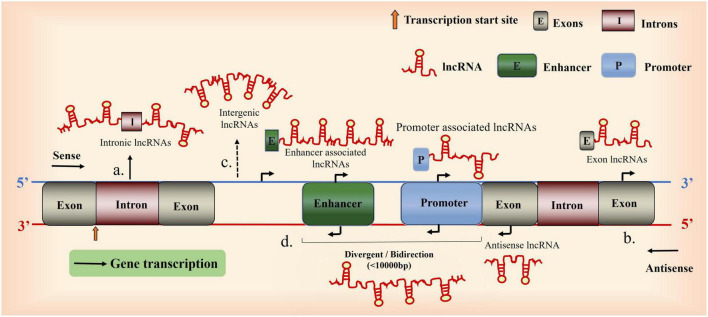
Overview of long non-coding RNA (lncRNA) biogenesis. A Schematic diagram illustrating the origin of lncRNAs. (a), Intron sense or antisense lncRNAs. (b), Exon sense- or antisense lncRNAs. (c), between genes. (d), Bidirectional lncRNAs (the coding transcript is initiated in a genomic region of less than 1,000 bp).

The functions of lncRNAs, which depend on their subcellular localization, vary in the cell membrane, cytoplasm, and nucleus. Bioinformatic analysis, subcellular localization experiments, and comparative analysis using databases enable the identification of lncRNA expression sites and corresponding functions (Mas-Ponte et al., 2017); lncRNAs are potential therapeutic targets for diseases. The neuroprotective and anti-neuroinflammatory effects of the lncRNAs nuclear-enriched abundant transcript 1(NEAT1) (Barry et al., 2017); and Gm4419, respectively, are dependent on their subcellular localization. The functions of lncRNAs are as follows: first, signaling molecules: signaling between different lncRNAs and cellular signal transduction (Flynn and Chang, 2014; Park et al., 2014); second, decoy molecules: regulate transcription by recruiting proteins or RNAs (Yan et al., 2015; Si et al., 2019; Peng et al., 2020); third, guidance molecules: bind specifically to chromatin-modifying proteins and regulatory sites and modulates the expression of upstream and downstream genes (Kim et al., 2012; Ferrè et al., 2016; Wang et al., 2016); finally, lncRNAs can function as a cellular scaffold, by binding and mediating the regulatory effects of specific proteins or RNAs (Modarresi et al., 2012; Zhang et al., 2019; Wang et al., 2020); lncRNAs mediate the epigenetic regulation of genes (DNA methylation, gene silencing, nucleolus dominance, histone modification, genomic blotting, maternal effects, dormant transposon activation, and RNA editing), as well as the transcriptional and post-transcriptional regulation of genes (RNA shearing, processing, splicing, metabolism, maturation, and stability) (Beermann et al., 2016; Matsui and Corey, 2017) (Figure 1).

Long non-coding RNAs were originally considered as simple transcriptional byproducts: “dark matter,” and “noise.” However, lncRNAs are involved in the regulation of various physiological and pathological processes, such as immunity, inflammation, gene regulation, and cell differentiation and proliferation (Mercer et al., 2009). In addition to TBI, lncRNA expression is dysregulated in neurodegenerative diseases (NDDs), ischemic cerebrovascular disease, glioma, and other neurological disorders (Xue et al., 2019). For example: In Parkinson’s disease, lncRNA NEAT1 increases stabilization of PTEN-induced putative kinase 1 (PINK1), hence promoting cellular quality control mechanism (Simchovitz et al., 2019). Recently, it was discovered that NEAT1 levels were significantly upregulated in the peripheral blood cells of PD patient (Boros et al., 2020). Amyotrophic Lateral Sclerosis (ALS) is prevalent form of motor neuron disease, Data have shown that NEAT1_2 was minimally expressed in the motor neurons of healthy people but highly expressed in the motor neurons of people with early-stage ALS, thus suggesting its utility as a biomarker for early ALS diagnostics (Zhang M. et al., 2021). LncRNAs are particularly attractive biomarkers because they are stable in the blood and other biofluids, and can be readily detected in easily-obtained biofluid samples. For example, some immune-related lncRNAs have been proposed as biomarkers and therapeutic targets for inhibiting glioma development. In TBI, dysregulated lncRNAs may regulate secondary brain injury through the mitogen-activated protein kinase signaling pathway, the p53 signaling pathway, or cytokine-receptor interactions (Patel et al., 2018). Exosomes, which are nanosized endocytic vesicles that are secreted by most cells, contain an abundant cargo of different RNA species that may be used as circulation biomarkers for diseases (Li et al., 2018), Here, we can browse Website access database query,^1^ exoRBase, which is a repository of circular RNA (circRNA), long non-coding RNA (lncRNA), and messenger RNA (mRNA) derived from RNA-seq data analyses of human blood exosomes. Determining the structure and function of lncRNAs is an active field in both genetics and cancer biology research, and several novel mechanisms and roles of lncRNAs have been reported. Improving our understanding of the function and expression of lncRNAs in health and disease and the underlying mechanisms, will provide novel insights that could be useful for disease prevention, non-invasive diagnosis, and predictive prognosis.

## Role of Inflammation and Immune Responses in Traumatic Brain Injury

Inflammation is the host defense response against invading pathogens and damaged cells under physiological conditions, which maintains tissue homeostasis (Sorby-Adams et al., 2017). However, inflammation can also mediate various pathological conditions, such as secondary brain injury. Thus, inflammation has a critical role in the development and alleviation of pathological conditions (Ghirnikar et al., 1998; Kaur and Sharma, 2018). Pathological conditions such as secondary brain injury aberrantly activate the inflammatory cascades, including microglial activation, peripheral leukocyte migration and recruitment, and cytokine release. This inflammatory response resembles a “gunpowder keg” (Merrill and Benveniste, 1996; Casili et al., 2018), which leads to blood-brain barrier (BBB) disruption, brain edema, exacerbation of brain injury (Corrigan et al., 2016), and systemic inflammatory response syndrome (SIRS). Post-injury inflammation and brain edema are key pathological events leading to secondary brain injury (Hopp et al., 1998). Trauma-induced oxidative stress promotes the release of pro-inflammatory factors and exacerbates inflammation through the activation of nuclear transcription factor-kappa B (NF-κB) (Yang et al., 2020) and activates its downstream mediator nucleotide-binding oligomerization domain (NOD)-like receptor protein 3 (NLRP3), which may regulate interleukin secretion. NLRP3 belongs to the innate immune response recognition receptor family, which plays an important role in TBI (Tang et al., 2020). Therefore, NLRP3 inhibition may mitigate the progression of neuroinflammation in TBI (Irrera et al., 2017).

Inflammation can exacerbate primary or secondary injuries in patients with TBI. Immunity refers to the recognition and response of the body’s immune system to self and danger signals intending to recognize and eliminate antigens and their foreign substances to maintain the physiological homeostasis and stability of the body. Guttman et al. (2009) reported that lncRNAs regulate the immune response. Both innate and acquired immunities are involved in TBI. During an immune response, ligands of the major histocompatibility complex (MHC)-associated T-cell receptor stimulate cytokine receptors in the presence of αβ T cells, which promotes the immune response in the CNS (Needham et al., 2019). At 2 h post-TBI, neutrophils accumulate at the site of central injury through the disrupted BBB (Soares et al., 1995; Rhodes, 2011). This leads to the activation of microglia, the infiltration of a large number of immune cells in the CNS, and the release of cytokines and chemokines. Cytokines and chemokines recruit various immune cells to the lesion site through G protein-coupled receptors. These immune cells secrete various cytokines, which initiate an inflammatory signaling cascade response that recognizes and attacks myelin sheaths and neurons and consequently exacerbates brain injury (McKee and Lukens, 2016; Needham et al., 2019). In contrast, activated microglia can be polarized toward a macrophage-like phenotype (called M1 type), which leads to the upregulation of NF-κB transcription and production of pro-inflammatory cytokines that can further promote the phagocytosis of extracellular debris and presentation of antigens to circulating lymphocytes (Benarroch, 2013). However, the activation of immune response exceeding the antigen-presentation flux leads to immune-mediated CNS damage. Additionally, the secretion of pro-inflammatory factors may activate other immunomodulators and exacerbate neuroinflammation.

The brain is termed as a “partial immune exempt organ.” In physiological conditions, the BBB prevents the entry of most metabolites and antigens into the brain. The neuronal metabolites and small molecular antigens of the self-circulatory system enter the brain tissue through areas lacking the BBB and the periphery through intracranial lymphatic drainage to maintain homeostasis (Absinta et al., 2017; Mondello et al., 2021); In contrast, pathological conditions, such as TBI are associated with the disrupted BBB. Additionally, pathogens and their metabolites expose concealed antigens, which can enter the skull and stimulate the brain to produce antibodies against the brain antigens, which bind to S1000 proteins, myelin base proteins, and neuron-specific enolases to form immune complexes that induce neuroimmune damage. The major cells involved in immune responses are microglia, macrophages, T and B lymphocytes, endothelial cells, mast cells, and natural killer (NK) cells (Jassam et al., 2017). The antigen-antibody complex can activate the complement system, which exerts complement-dependent cytotoxic effects and consequently promotes neuronal apoptosis (Wei et al., 2020). Antibodies against brain antigens stimulate microglia to overexpress IL-1, which induces neuronal apoptosis by activating the transcription and translation of the apoptotic gene CASP3. Additionally, the anti-brain antigen antibodies attack the brain tissue, which results in the exacerbation of the inflammatory response, generation of large amounts of oxygen free radicals, induction of lipid peroxidation in the neuronal cytoplasm, impairment of mitochondrial metabolism, and the induction of cerebral hypoxia and cerebral edema. The secreted neurotransmitters, such as excitatory amino acids, interact with neurons and astrocytes and increase the levels of Ca^2+^ and Na^+^, thereby disrupting the BBB, promoting cytotoxic brain edema (Dixon, 2017; O’Leary and Nichol, 2018) and exacerbating brain injury. Therefore, the suppression of inflammation is a potential therapeutic strategy for TBI. Glucocorticoids, which are immunosuppressive drugs, have been regarded as therapeutic agents for vascular brain edema. However, the effectiveness of glucocorticoids is dependent on the type of brain edema. The major cause for cytotoxic edema is damage to cell membrane ion channel, while that for vascular-derived edema is the disruption of BBB and altered vascular permeability. Although glucocorticoids exert anti-inflammatory effects, they are not recommended for patients with severe TBI to suppress immunity or alleviate brain edema as it can lead to increased mortality (Carney et al., 2017).

### Role of Long Non-coding RNA-Mediated Immune Responses in Traumatic Brain Injury

The expression profiles of lncRNAs, which are differentially expressed in the CNS, are correlated with specific neuroanatomical regions and cell types (Khorkova et al., 2015). Some studies have reported that lncRNAs mediate inflammation after TBI by regulating different inflammatory pathways, which further exacerbates CNS injury (Simon et al., 2017); The major pathophysiological process underlying TBI are neuroinflammation, apoptosis, axonal disruption, regeneration, and angiogenesis, which may be related to the regulation of lncRNAs (Zhang and Wang, 2019). For example, various lncRNAs, such as NEAT1, MALAT1, maternally expressed gene 3 (MEG3), and growth arrest-specific transcript 5 (GAS5) regulate inflammatory pathways and immune responses that mediate apoptosis in neuronal cells (Zhang and Wang, 2019). Additionally, oxidative stress plays an important role in mediating secondary injury after TBI (Carrillo-Vico et al., 2014; Miao and Liang, 2018). lncRNA-mediated inflammatory responses after TBI cause damage not only to the brain, but also to other organs and systems (e.g., SIRS, neurogenic pulmonary edema, Cushing’s ulcer, and endocrine changes). Therefore, the effective regulation of lncRNA expression can alleviate neuroinflammatory injury and consequently mitigate primary and secondary brain injuries in patients with TBI.

Long non-coding RNA play an important role in the development, proliferation, and differentiation of intrinsic and adaptive immune cells (Wu et al., 2015). Dendritic cells (DCs) are potent antigen-presenting cells. Conventionally, DCs were not considered to be localized to the CNS in which microglia and peripherally derived monocytes/macrophages served as the primary antigen-presenting cells. However, recent studies have reported that DCs are localized to the CNS and are actively involved in injury response (D’Agostino et al., 2012); DCs and monocytes/macrophages are involved in intrinsic immunity. Previous studies examining the role of lncRNAs in DC differentiation and function have identified specific lncRNAs that are upregulated in DCs (termed lnc-DCs) can regulate DC differentiation, prevent their accumulation, and mitigate excessive inflammatory responses (Wang et al., 2014); For example, lnc-DCs can directly bind to signal transducer and activator of transcription 3 (STAT3) in the cytoplasm and suppress inflammation by inhibiting the binding of STAT3 to SHP1 and dephosphorylating SHP1. Lnc-DC cannot effectively initiate the secretion of inflammatory factors from CD4+ T cells under DC-deficient conditions (Wang et al., 2019). In contrast, adaptive immunity, which has a “memory” function, is activated upon the stimulation of specific T and B lymphocytes by antigens during biological evolution. Previous studies on acquired immunity have reported that lncRNA MAF-4, which is a chromatin-associated Th1-specific lncRNA, was negatively correlated with the expression of the Th2-associated transcription factor MAF. The downregulation of lncRNA MAF-4 resulted in the differentiation of T cells to Th2 cells (Ranzani et al., 2015). The functions of NK cells, which have been derived from the bone marrow, are not dependent on antibodies. Intrinsic NK cells in the brain inhibit Th17 cell activation and consequently attenuate the inflammatory response, demyelination, and immune cell infiltration in the brain (Hao et al., 2010, 2011); This suggested that intrinsic and adaptive immunities may have overlapping functions. However, the specific mechanisms and regulation of adaptive immunity have not been completely elucidated. Hence, the effective alleviation of inflammation has not been achieved to reverse the associated cascade damage and improve the prognosis of TBI.

Therefore, lncRNAs mediate inflammatory responses in TBI and play an important role in secondary brain injury. Different lncRNAs can exert similar cerebral protective effects through the same pathway or different pathways. Hence, there is a need to explore the inflammatory pathways that are dysregulated during TBI and how lncRNAs can interact with these pathways. The next section will focus on the role and regulatory mechanisms of specific lncRNAs in TBI, which could aid the development of novel strategies for the diagnosis, treatment, and prognosis of patients with TBI.

### Correlation of Different Long Non-coding RNAs With Inflammation in Traumatic Brain Injury

#### Long Non-coding RNAs Metastasis-Associated Lung Adenocarcinoma Transcript 1

Metastasis-associated lung adenocarcinoma transcript 1, which was first discovered in 2003, is located on the long arm of chromosome 11 (11q13.1). The characteristics of MALAT1 are as follows: length, 8,700 nt; expression site, predominantly the nucleus; lacks a well-defined open reading frame (Liu et al., 2010). MALAT1 regulates the distribution and activity of serine/arginine (SR) splicing factors, while SR regulates variable splicing of pre-mRNAs by promoting phosphorylation. Therefore, MALAT1 is also known as nuclear-enriched autosomal transcription product 2 (NEAT2) (Zhang et al., 2017b; Statello et al., 2021). MALAT1 is specifically cleaved into a large 5’ terminal fragment of a non-coding fragment containing a polyA tail in the nucleus and a small 3’ terminal long tRNA-like fragment in the cytoplasm (Arun and Spector, 2019).

Long non-coding RNAs metastasis-associated lung adenocarcinoma transcript 1 is known as a “star molecule” that regulates key biological processes, such as cell proliferation and differentiation and may be involved in regeneration after brain injury (Tajiri et al., 2014; Ghattas et al., 2016; Zhang et al., 2017d). Additionally, MALAT1 is associated with the distant metastasis and prognosis of non-small cell lung cancer (Ji et al., 2003). MALAT1 expression is upregulated in various cancer and paraneoplastic tissues and that the expression is closely correlated with tumor size and envelope invasion. In TBI, MALAT1 exerts neuroprotective effects and is involved in the regulation of mRNA splicing, editing, and stability. Previously, exosomes from human adipose stem cells were used as a therapeutic for TBI. The exosomes increased the number and proliferation of neurons, which can be attributed to multiple regulatory therapeutic targets of MALAT1. MALAT1 improved the activity of damaged neurons through different pathways. *In vitro* experiments with mouse hippocampal neuronal cells (HT22) reported that MALAT1 exerts neuroprotective effects. Studies on brain injury models have demonstrated that the neuroprotective effect of exosomes is mitigated upon depletion of MALAT1 in the exosomes (Ghattas et al., 2016). The release of immune cells from the spleen and their infiltration into the brain is induced at the onset of TBI, which exacerbates secondary brain injury (Stewart and McKenzie, 2002). However, the inhibition of monocyte infiltration into the brain alleviates secondary injury and promotes the recovery of cognitive function (Morganti et al., 2015). The spleen is an important source of peripheral macrophages and monocytes. Treatment with exosomes may inhibit the release of these immune cells into the circulation, reduce immune cell infiltration into the brain *via* the disrupted BBB, and mitigate the peripheral immune cell-mediated exacerbation of secondary injury. Thus, MALAT1 is a potential therapeutic target for TBI.

Overexpression of lncRNA MALAT1 significantly inhibited brain edema in a rat model of TBI and downregulated the expression of IL-6, NF-κB, and aquaporin 4 (AQP4). MALAT1 expression is upregulated in monocytes of immune disease models [e.g., systemic lupus erythematosus (SLE)] (Yang et al., 2017). The role of MALAT1-mediated inflammation in TBI has not been reported. However, MALAT1-mediated immune responses may be involved in the pathogenesis of secondary cranial brain injury. Further studies are needed to examine the role of MALAT1 in exacerbating or protecting cerebral injuries. The cerebral protective effect of lncRNA MALAT1 is higher than the cerebral injury exacerbation effect. Additionally, MALAT1 exerts an overall protective effect. However, the underlying mechanisms must be further investigated. Astrocytes are more prone to TBI-induced damages than other neuronal cells (Burda et al., 2016). MALAT1 is downregulated in the rodent fluid percussion injury model of TBI, which is consistent with brain edema and astrocyte swelling and may be related to AQP4 expression and BBB disruption. The diagnostic and therapeutic potentials of MALAT1 in other diseases have been demonstrated. MALAT1 induces autophagy and consequently protects the brain microvascular endothelial cells against oxygen-glucose deprivation (OGD)/reoxygenation injury (Li et al., 2017). Li et al. (2017) reported that MALAT1 suppressed cerebrovascular endothelium-induced apoptosis during cerebral ischemia-reperfusion injury (IRI). The expression levels of MALAT1 and inflammatory factors (TNF-α and IL-1β) were significantly upregulated in the spinal cord injury model. Additionally, the level of microglial activation was significantly and positively correlated with MALAT1 expression. MALAT1 activates the downstream IKKβ/NF-κB signaling pathway through the downregulation of miR-199b, which promotes the release of inflammatory factors from the microglia (Zhou et al., 2018). Microglia have an important role in the regulation of CNS development, maturation, and degeneration and its microenvironment. Additionally, optimal inflammatory responses protect the neurons against brain injury. However, sustained activation of immune responses promotes the release of inflammatory factors, which induce neuronal necrosis (Wang and Zhou, 2018).

#### Long Non-coding RNA Nuclear Enriched Abundant Transcript 1

Nuclear enriched abundant transcript 1 (4,000 bp), which is involved in the formation of paraspeckles, is encoded by a region located on the long arm (11q13) of the multiple endocrinopathies type I gene on human chromosome 11 locus (Klec et al., 2019). Additionally, NEAT1 encodes NEAT1-v1 (3.7 kb) and NEAT1-v2 (23 kb), which are localized in the paraspeckles and share the same promoter but their 3’ ends are processed by different mechanisms. The function of paranuclear vesicles is unclear. Some studies have reported the correlation between the paranuclear vesicles and mammary gland formation, immune diseases, and bone marrow myeloid differentiation. Paraspeckles contain several factors, such as SFPQ, p54nrb, and RBN14. NEAT1 promotes paranuclear vesicle formation by directly binding to these protein factors. The knockout or knockdown of NEAT1 leads to the disruption of paraspeckles (Gast et al., 2019). SFPQ inhibits IL-8 expression in unstimulated cells by binding to the promoter region of chemokine 8 (CXCL8) (Ahmed and Liu, 2018); NEAT1 plays an important role in intrinsic immunity by upregulating the expression of inflammatory factors in stimulated cells. Bioinformatics and experimental analyses revealed that NEAT1 can negatively regulate the expression of miR-365, which can modulate the downstream BAX and AKT pathways and promote apoptosis. However, further studies are needed to examine NEAT1-mediated neuroinflammation.

Nuclear enriched abundant transcript 1 exhibits differential expression in TBI (Zhong et al., 2016). At 24 h post-TBI, NEAT1 expression is significantly upregulated (by approximately three-fold) in the cerebral cortex at the injury site. TBI results in varying degrees of axonal damage. The damaged axons regenerate and elongate to restore neuronal function, a process coordinated by several key factors (Pajeau, 2001). Axon elongation markedly increased upon NEAT1 overexpression (Zhong et al., 2017). Conversely, axon elongation was significantly suppressed upon NEAT1 knockout. These findings provide novel insights into the mechanisms regulating axon elongation at the molecular level. Recent studies has reported that p53-inducible protein 1 (PIDD1) is involved in both pro-apoptotic and anti-apoptotic pathways depending mainly on the type and severity of the injury (Bock et al., 2012). PIDD1 exerts a pro-apoptotic effect on HT22 cells subjected to OGD stress and affects neurons in the edema zone after TBI. The pro-apoptotic effect of PIDD1 has been suppressed upon lncRNA NEAT1 overexpression. PIDD1 enhanced the functional recovery of neurons after TBI. The downregulation of NEAT1 suppressed IL-6, IL-1β, and TNF-α expression in controlled cortical impact (CCI) rats, alleviated neuroinflammation (Xia et al., 2018) and improved the prognosis.

Dysregulation of NEAT1 promotes the development of various diseases, such as viral infections, neurodegenerative diseases, autoimmune diseases, and cancer (Prinz et al., 2019). The role of NEAT1 in immune-related diseases involves regulating paranuclear spot-mediated gene expression, which is consistent with the function of the paranuclear spot mentioned above. Studies to elucidate the structure and functions of NEAT1, such as the nuclear retention mechanisms of RNA or transcription of immune regulatory genes (Imamura and Akimitsu, 2014; Imamura et al., 2014). Previous studies on SLE revealed that NEAT1 expression was upregulated in monocytes and positively correlated with SLE disease severity. Conversely, silencing NEAT1 significantly downregulated the expression of cytokines (such as IL-6) and CXCL10 (Zhang F. et al., 2016); NEAT1 promotes brain injury in septic mice by positively regulating NF-κB in B cells, which was attenuated upon transfection with si-NEAT1 (Barry et al., 2017). Additionally, NEAT1 protects the cells against lethal injury and prevents early apoptosis after TBI, which suggested that NEAT1 regulates apoptosis (Hirose et al., 2014). Primary pathological changes in TBI are mainly manifested in the brain and include tissue contusion and neuronal axon disruption. NEAT1 upregulation promotes neuronal axon repair and growth. Additionally, NEAT1 upregulation promotes the release of cytokines and chemokines, which adversely affect the injured neurons. Chemokines promote the accumulation of peripheral inflammatory cells at these sites. Several studies have focused on the advantages and disadvantages of increased neutrophils after TBI and suggested that the effects of neutrophils depend on the degree of TBI and the expression of si-NEAT1 after TBI. Severe TBI exacerbates metabolic disorder and cerebral perfusion deficit even if the neuroprotective mechanism of si-NEAT1 is dominant and its expression is not marked. The structure of paranuclear vesicles (paraspeckles) and the expression of NEAT1 must be examined in future studies.

#### Long Non-coding RNA Gm4419

Gm4419, which was originally identified in the kidney of mice with diabetic nephropathy, exhibits pro-inflammatory properties in renal inflammation and fibrosis. Gm4419 is located on chromosome 12 (Chr12:21417911-21419803) and has a length of 1,730 bp (Ensembl ID: ENSMUST00000180671) (Yi et al., 2017).

The onset of TBI leads to the elicitation of local and systemic inflammation (Kumar and Loane, 2012; Kumar et al., 2017). Aberrant inflammatory response, which is an important pathological mechanism of secondary brain injury, promotes neuronal loss, neurological deficits, brain edema, glial activation, and release of inflammatory mediators (Loane and Faden, 2010). Gm4419 is aberrantly upregulated *in vitro* after astrocyte injury. The overexpression of Gm4419 in injured astrocytes upregulated the expression of TNF-α, BAX, cleaved CASP3 and CASP9, and downregulated BCL-2 and CyclinD1 levels and promoted apoptosis. Conversely, the knockout of lncRNA Gm4419 exerted the opposite effects. Mechanistically, Gm4419 transcripts can act as a sponge for miR-466l and miR-466lco and promotes trauma-induced astrocyte apoptosis by upregulating inflammatory cytokines. Thus, Gm4419 can upregulate TNF-α expression by competitively binding to miR-466l, which subsequently promotes inflammatory injury. Gm4419 can activate the NF-κB pathway by directly interacting with p50, a subunit of NF-κB, and caspase, a component of the constituent inflammasome NLRP3. Thus, Gm4419 co-mediates glial cell apoptosis by promoting inflammation, fibrosis, and proliferation after TBI through the NF-κB/NLRP3 signaling pathway (Xue et al., 2019). IRI is critical for secondary brain injury. lncRNA Gm4419 is upregulated in the IRI rat model and the knockout of lncRNA Gm4419 alleviates apoptosis (Ying et al., 2020).

The immune mechanism of Gm4419 in TBI has not been completely elucidated, which can be attributed to its physicochemical properties. The role of Gm4419 in inflammation must be further investigated in future studies. Thus, lncRNA Gm4419 plays an important role in TBI by mediating the pathological inflammatory process and can be a potential therapeutic target for TBI (Yu et al., 2017).

#### Long Non-coding RNA Growth Arrest-Specific Transcript 5

Growth arrest-specific transcript 5 (4,983 bp), which was originally identified in cells exhibiting growth arrest, is located on human chromosome 1q25.1 and contains multiple introns and exons. The exons express the GAS5 RNA sequences, while the intron sequences encode 11 snoRNAs involved in rRNA production (Mayama et al., 2016). Although the exons contain an open reading frame, they do not encode functional proteins (Lu et al., 2013). The continuous accumulation of GAS5 in growth-arrested cells is associated with attenuated translation and suppressed degradation of mammalian target of rapamycin (mTOR) and 5’TOP RNA, which leads to a marked accumulation of spliced, mature lncRNA GAS5 (Schneider et al., 1988). Activated p53 protein, which induces p21 expression under uncontrolled cell division and proliferation conditions, promotes cell cycle arrest and GAS5 upregulation. p53 is expressed when cells undergo irreparable damage through the induction of pro-apoptotic genes (e.g., BAX, a BCL-2 family member). GAS5, p53, and p21 form a mutually reinforcing triangle that accelerates programmed cell death (Kanapathipillai, 2018; Ni et al., 2019).

Recent studies have reported that GAS5 is dysregulated in autoimmune diseases and cancer (Pickard et al., 2013; Gao et al., 2018). GAS5 regulates apoptosis, mediates cell survival, and influences metabolic activity (Shi et al., 2015). Enhanced intracranial pressure after TBI can induce oxidative damage (Liu et al., 2007). GAS5 negatively regulates miR-124, which is regulated by oxidative stress. miR-124 downregulation after TBI leads to the overproduction of reactive oxygen species, whereas miR-124 upregulation promotes cell survival (Zhang et al., 2017c), this suggested that GAS5 knockdown attenuates oxidative stress injury by upregulating miR-124 (Miao and Liang, 2018). More than 50% of patients with severe TBI develop severe brain injury, which is characterized by neuronal cell damage resulting from a complex series of endogenous pathophysiological processes triggered after primary brain injury (Butcher et al., 2007). TBI-activated microglia release high levels of pro-inflammatory factors (e.g., interferon-γ, TNF-α, and IL-1β), chemokines, and ROS, which leads to chronic neuroinflammation, oxidative stress and neurodegeneration, and inhibition of neural regeneration (Xu et al., 2018). Mitochondria are widely distributed in the neuronal axons, terminals, and dendrites. Traumatic insult to the brain tissue leads to the dysregulation of local metabolism, enhanced production of ROS, accumulation of lipid peroxides on mitochondrial membranes, severe impairment of energy metabolism, and consequently neuronal degeneration and apoptosis.

Therefore, silencing GAS5 may reduce the incidence of brain injury and neurodegenerative diseases by mitigating oxygen free radical formation after TBI and decreasing the accumulation of immune complexes.

#### Long Non-coding RNA Maternally Expressed Gene 3

Maternally expressed gene 3 (1,700 nt), which is located on human chromosome 14q32.3 (Braconi et al., 2011), can be selectively spliced to produce 12 different MEG3 transcripts (Mondal et al., 2015). MEG3a, MEG3b, MEG3c, and MEG3d are four isoforms of MEG3 and each isoform contains one exon. In total, 12 cDNA isoforms, named MEG3, MEG3a, and MEG3b–k (GQ183494–GQ183503), were identified using real-time polymerase chain reaction (Binabaj et al., 2018).

Maternally expressed gene 3 is reported to be involved in tumor suppression, recent studies have demonstrated that MEG3 can regulate ischemic neuronal death and exert neuroprotective effects (Yan et al., 2017). Injury is a multisystem lesion involving complex interactions between neural, immune, and peripheral systems (Saber et al., 2020). TBI disrupts the integrity of the BBB, which separates the brain parenchyma from blood (Jing et al., 2020). The inflammatory response following TBI is caused by the entry of peripheral immune mediators through the damaged BBB (Sivandzade et al., 2020). Microglia are reported to be involved in the neuroinflammatory and immune responses to TBI (Bedi et al., 2018). MEG3 is expressed in several human tissues, including the brain and adenohypophysis. Additionally, MEG3 may be involved in neuronal remodeling and is essential for neuronal resistance to injury (Liu et al., 2017). miR-548d-3p can regulate the JAK-STAT signaling pathway through inhibition of SOCS5 and SOCS6 expression by binding to the 3’UTR of the suppressor of cytokine signaling. MEG3 upregulation accelerates neuronal apoptosis through this pathway (Tan et al., 2019). Bioinformatics analysis and dual-luciferase experiments have revealed that MEG3 directly targets and downregulates miR-7a-5p expression. miR-7a-5p suppresses the effect of MEG3 upregulation on microglial activation. MEG3 functions as a miR-7a-5p competitive endogenous RNA that induces microglial inflammation by regulating NLRP3 expression.

Thus, the Meg3/miR-7a-5p/NLRP3 axis is a potential therapeutic target for TBI (Meng et al., 2021).

#### Long Non-coding RNA Colorectal Neoplasia Differentially Expressed

Long non-coding RNA colorectal neoplasia differentially expressed (CRNDE) (1,059 bp), which is located on human chromosome 16 (16q12.2) adjacent to IRX5, contains five core exons. Previous studies have reported that CRNDE is a proto-oncogene that is expressed in gliomas and that its expression is negatively correlated with the prognosis of tumor (Gao et al., 2017; Shi et al., 2017; Zhang et al., 2020). Lin et al. (2011) reported that CRNDE is expressed during the neural differentiation of human-induced pluripotent stem cells and that CRNDE regulates glioma development by promoting inflammation (Thermozier et al., 2020). CRNDE upregulation triggers WI-38 cell injury, significantly inhibits cell viability, and increases cytokine levels, which leads to the induction of inflammation and apoptosis (Yang Y. P. et al., 2018).

Colorectal neoplasia differentially expressed is upregulated in TBI (Yi et al., 2019). The knockdown of CRNDE suppressed neuroinflammatory factor expression, improved neurobehavioral function in TBI rats, upregulated the levels of glial fibrillary acidic protein (GFAP), bromodeoxyuridine nucleoside, nerve growth factor, and nestin, and suppressed neuronal apoptosis and autophagy. These changes lead to the induction of neuronal differentiation and nerve fiber growth and regeneration (Yi et al., 2019). Neuroinflammation after TBI may exacerbate neurological dysfunction, tissue damage, and neuronal loss (Byrnes et al., 2012; Breunig et al., 2013). Elevated pro-inflammatory cytokines, immune cell proteases, and oxidative stress after TBI can lead to excessive tissue damage and induction of neuronal cell death (Stover et al., 2000; Rothwell, 2003). Additionally, the upregulation of reactive astrocytes and GFAP are considered to be markers of CNS injury (Kernie et al., 2001).

**FIGURE 2:**
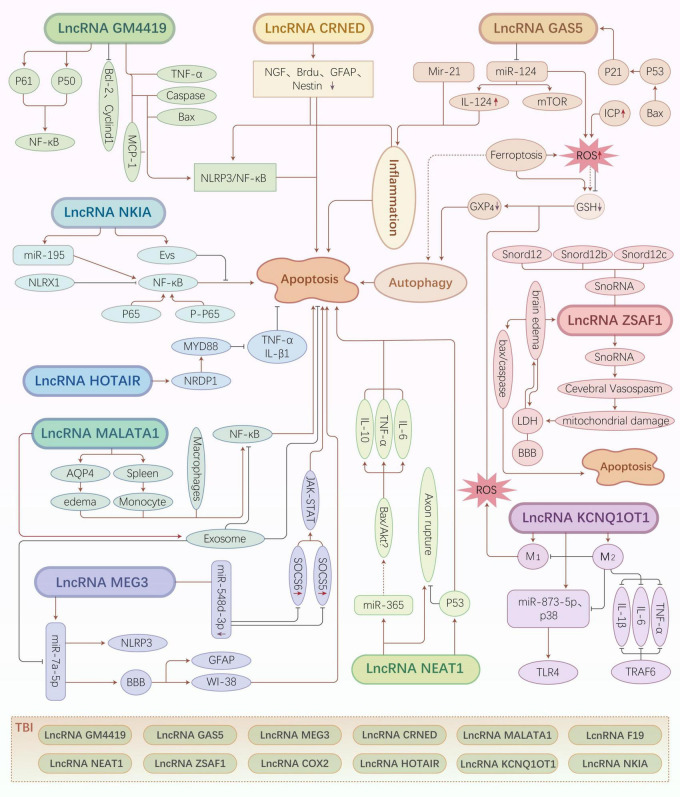
Traumatic brain injury (TBI)-related long non-coding RNA (lncRNA) regulatory mechanisms and pathways. Different types of lncRNAs regulate various downstream molecules that exert neuroprotective or neuroapoptotic effects by inducing cell death pathways, such as inflammatory pathways, oxidative stress, autophagy, and ferroptosis.

Colorectal neoplasia differentially expressed is closely related to neuroinflammation. The downregulation of CRNDE exerts a protective effect against neuronal damage. Hence, the effective regulation of CRNDE expression may alleviate TBI. However, further studies are needed to elucidate the underlying regulatory mechanisms or pathways (Figure 2).

#### Long Non-coding RNA HOX Transcript Antisense RNA

HOX transcript antisense RNA (2,148 nt) was first identified by Rinn et al. (2007). Human HOTAIR is located at the HOXC locus on chromosome 12q13.13 flanking between HOXC11 and HOXC12.

HOX transcript antisense RNA plays an important role as a star molecule in several diseases, especially tumors and immune diseases. For example, in rheumatoid arthritis, overexpression of lncRNA HOTAIR significantly suppressed NF-κB in chondrocytes treated with lipopolysaccharide by inhibiting the nuclear translocation of p65 and consequently downregulated the IL-1β and TNF-α levels (Zhang et al., 2017a). The study demonstrated that HOTAIR functions as a skeleton molecule and that the two ends bind to different histone modification complexes. The 5’ end binds to the PRC2 complex to exert a pro-methylation effect, while the 3’ end binds to the LSD1/CoREST/REST complex to exert a demethylation effect.

The expression of HOTAIR in microglia, which is activated in TBI, is upregulated in activated microglia. HOTAIR inhibits microglial activation and inflammatory factor secretion through Ki-67 and promotes neuromodulin receptor degradation protein (NRDP1)-mediated ubiquitination of myeloid differentiation factor (MYD88) protein (Cheng et al., 2021). Previous studies have reported that MYD88 expression is closely related to neurological function in mice with TBI and that MYD88 downregulation attenuates TBI by significantly suppressing the release of the glial cell activation marker IBA-1 and the inflammatory factors (TNF-α and IL-1β) (Zhang H. S. et al., 2016; Cheng et al., 2021). The inhibition of HOTAIR mitigated the progression of brain injury by inhibiting inflammatory pathways, which suggested the critical role of HOTAIR in TBI-induced inflammatory responses in microglia although the underlying mechanisms are not clear.

Granulocytes and macrophages are involved in the HOTAIR-induced immune response to TBI. Inflammation and immune response are often complementary processes that are not easily distinguishable. Previous findings indicate that HOTAIR exerts neuroprotective effects through multiple factors. However, the underlying mechanisms must be examined in future studies.

#### Long Non-coding RNA Zinc Finger X-Chromosomal Protein Antisense 1

Zinc finger X-chromosomal protein antisense 1 (ZFAS1) is derived from the antisense transcription of zinc-containing ferredoxin-1 (ZFX1) located on chromosome 20q13.13 (Dong et al., 2018). Previous studies have reported that ZFAS1 is associated with the development and progression of various cancers (Feng et al., 2021). ZFAS1 contains snoRNAs (Snord12, Snord12b, and Snord12c) (Askarian-Amiri et al., 2011).

The expression of ZFAS1 is upregulated in TBI mice (Wang et al., 2017). The knockdown of ZFAS1 inhibited apoptosis and inflammation and attenuated brain edema and neuronal injury (Feng et al., 2021). Brain edema, an important prognostic factor, is a key challenge in the treatment of TBI (Winkler et al., 2016). The inactivation of ZFAS1 downregulates the levels of BAX and cleaved CASP3 (Zhao et al., 2019), inflammatory factors and neuronal cell death. Clinically, patients with TBI often have injuries at sites in addition to the head, such as the neck or spinal cord in the event of a whip-like injury or an injury resulting after a fall from height. In mice with spinal cord injury (SCI), ZFAS1 is upregulated in the spinal cord tissue. Additionally, the knockdown of ZFAS1 promoted functional recovery and inhibited neural cell apoptosis and inflammatory response (Chen et al., 2021).

Therefore, ZFAS1 has a critical role in CNS injury. The knockdown of ZFAS1 alleviates secondary brain injury and SCI but the underlying regulatory mechanisms have not been elucidated. There is a need to elucidate the pathways and mechanisms of ZFAS1 that mediate the pathogenesis of diseases to improve the clinical treatment and prognosis of the disease.

#### Long Non-coding RNA NF-κB-Interacting Long Non-coding RNA

NF-κB-interacting long non-coding RNA (NKILA) was first identified and named by Liu et al. (2015), in 2015 based on breast cancer studies. NKILA (2,570 nt), which is located on chromosome 20q13, regulates the NF-κB signaling pathway (Lu et al., 2018). NKILA comprises a specific site for binding to NF-κB at 104–195 bp upstream of the transcription start site (Yu et al., 2018). Previous studies have demonstrated that NKILA regulates laryngeal cancer cell migration and invasion and that it is a potential clinical and prognostic biomarker for breast and esophageal cancers (Lu et al., 2018; Yang T. et al., 2018).

NF-κB-interacting long non-coding RNA, which negatively regulates NF-κB, can inhibit the activation of the NF-κB signaling pathway that is associated with the inflammatory response after TBI (Ke et al., 2018; Tao et al., 2018). NLRX1 can exert an inhibitory effect on secondary brain injury through the negative regulation of NF-κB signaling. Various lncRNAs and miRNAs exhibit differential expression in TBI (Zhong et al., 2016). Additionally, these lncRNAs and miRNAs exhibit both differential and overlapping functions. Extracellular vesicles (EVs) from pluripotent mesenchymal cells promote the recovery of neuronal cell function in TBI rats by upregulating endogenous angiogenesis and neurogenesis and downregulating inflammation (Zhang et al., 2015). NKILA delivered through EVs derived from astrocytes inhibits neuronal injury and promotes structural and functional recovery of the brain after TBI by upregulating miR-195, which targets NLRX1 (He et al., 2021). Astrocytes are close to neurons and provide nutrition and support to neurons (Lafourcade et al., 2016). EVs released from astrocytes and harboring NKILA are phagocytosed by neurons. In the neurons, NKILA exerts therapeutic effects (Rufino-Ramos et al., 2017). Additionally, studies on rat brain hemorrhage revealed that NKILA expression was downregulated in the cerebral hemorrhage group. Additionally, the expression levels of IκBα, p-IκBα, P65, and p-P65 were upregulated and the NF-κB signaling pathway was activated (Huang et al., 2018; Jia et al., 2018). Furthermore, the expression of inflammatory factors downstream of this pathway was upregulated, which leads to neuroinflammation.

Thus, NKILA and NLRX1 negatively regulate the NF-κB signaling pathway. The downregulation of NLRX1 expression and the inhibition of the NF-κB signaling pathway can mitigate neuroinflammatory damage after brain injury (Theus et al., 2017), which can improve the prognosis of patients with TBI.

#### Long Non-coding RNA Potassium Voltage-Gated Channel Subfamily Q Member 1 Overlapping Transcript 1

Potassium voltage-gated channel subfamily Q member 1 overlapping transcript 1 (KCNQ1OT1), which is located at the 11p15.5 locus (Kanduri, 2011) and has a length of 91,671 nt, is species homology and catalyzed by RNA polymerase II (Zhang K. et al., 2021).

Cytokines released from activated microglia continuously promote neuronal damage (Corrigan et al., 2016). Microglial activation is associated with the following two phenotypes: pro-inflammatory neurotoxic (M1) and anti-inflammatory neuroprotective (M2) phenotypes. The transition from M1 phenotype to M2 phenotype may play a beneficial role in TBI (Xu et al., 2017; Su et al., 2020). Previous studies have examined the role of KCNQ1OT1 in TBI development (Zhong et al., 2016). Various studies have demonstrated that lncRNAs exert pro-angiogenic, anti-apoptotic, and anti-inflammatory effects in TBI by regulating different molecules and pathways (Zhang and Wang, 2019). Gene chip analysis has revealed that several lncRNAs are differentially expressed in TBI rats, which may be related to TBI-induced inflammation, cell cycle, apoptosis, and other pathological processes (Wang et al., 2017). The knockdown of KCNQ1OT1, which mediates neuroinflammation, attenuates PC12-induced inflammation, oxidative stress, and apoptosis in the OGD model (Yi et al., 2020). KCNQ1OT1 is upregulated in the TBI mouse model and regulates the TLR4 pathway through the KCNQ1OT1/miR-873-5p axis. Additionally, KCNQ1OT1 knockdown significantly attenuates the expression of TLR4, MYD88, and TRAF6, and inactivates p38 and NF-κB phosphorylation (Di Padova et al., 2018). Furthermore, miR-873-5p inhibition mitigated the KCNQ1OT1 knockdown-mediated effects. TRAF6 is a direct target of miR-873-5p.

Potassium voltage-gated channel subfamily Q member 1 overlapping transcript 1 decreases BBB permeability, promotes the polarization of M1 toward M2, alleviates IL-1β, IL-6, and TNF-α-mediated inflammatory damage, reduces neuroinflammation in TBI mice, and exert neuroprotective effects by regulating the miR-873-5p, TRAF6, P38, and NF-κB pathways (Liu et al., 2021).

#### Long Non-coding RNA Cyclooxygenase 2

Cyclooxygenase 2 (COX2) [also known as prostaglandin-endoperoxide synthase 2 (PTGS2)], which was first identified 12 years ago, regulates immune response. lncRNA COX2 is located 51 kb upstream of COX2 where the lncRNA is significantly and differentially expressed and was therefore named lncRNA COX2 (gene ID: 854622) (Guttman et al., 2009).

Recent studies have demonstrated that lncRNA COX2 is localized in the nucleus and cytoplasm of cells and plays an important role in the primary cellular immune response. The induction of Toll-like receptors (TLRs) by macrophages promotes the expression of lncRNA COX2, which mediates the activation and suppression of immune-related genes in intrinsic immune cells (Carpenter et al., 2013). The activation of intrinsic immunity has an important role in the progression of TBI. lncRNA COX2 directly binds to NF-κB and promotes the nuclear translocation of p65. NF-κB is activated after brain injury. CASP1-processed IL-1β promotes neuroinflammation (Lalor et al., 2011). lncRNA COX2 knockout inhibits NLRP3 inflammatory vesicles by downregulating the mRNA and protein levels of NLRP3 and ASC, which inhibit caspase-1 activation. Additionally, lncRNA COX2 knockout-mediated NLRP3 downregulation attenuates inflammasome activation, which suppresses pro-inflammatory IL-1β secretion by macrophages and microglia *in vitro* (Xue et al., 2019). lncRNA COX2 expression may be dependent on MYD88 ubiquitination and NF-κB signaling pathways. Therefore, lncRNA COX2 inhibits microglial activation *via* ubiquitination and consequently attenuates the TBI inflammatory response.

The role of lncRNA COX2 in TBI is unclear. However, cytokines, inflammatory vesicles, and immune-related responses are key factors in the inflammatory cascade response in secondary brain injury and play an important role in the development of TBI. As lncRNA COX2 regulates the levels of these factors, it may also mediate the pathogenesis of TBI. Further studies are needed to examine the interactions between lncRNA COX2 and immune mediators and pathways and the underlying mechanisms.

#### Long Non-coding RNA F19

**FIGURE 3:**
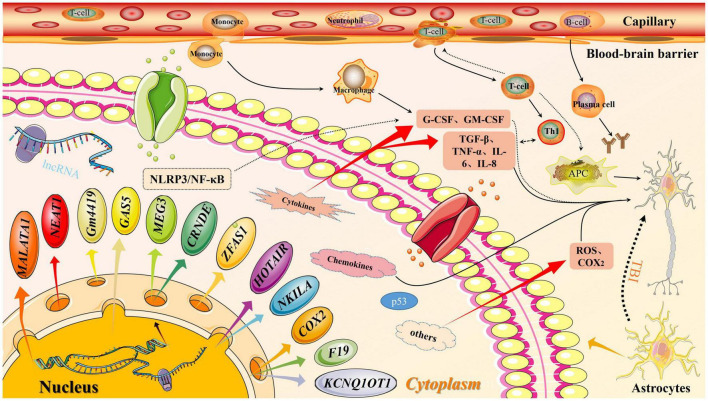
Long non-coding RNA (lncRNA) molecular pattern diagram in traumatic brain injury (TBI). In traumatic brain injury, the expression of lncRNA downstream molecules increases, which activates the inflammation pathway. At the same time, the permeability of the blood-brain barrier increase, thereby releasing inflammatory factors. Both factors will affect secondary brain injury.

Other lncRNAs, such as lncRNA F19 are reported to be involved in TBI. The overexpression of lncRNA F19 can exacerbate secondary brain injury through the TLR4 pathway. Conversely, the inhibition of lncRNA F19 overexpression can mitigate neuronal apoptosis through the same pathway (Peng et al., 2019). The role of F19 in other diseases, including brain injury, and immune inflammation and the underlying mechanisms are unclear. The development of molecular technologies and novel experimental designs may enable the elucidation of specific mechanisms of various lncRNAs in diseases (Figure 3).

## Treatment of Traumatic Brain Injury-Associated Inflammatory Injury and Immune Response

Clinically, TBI patients are often escorted to the hospital urgently. Thus, the specific course of injury in patients with TBI is rarely monitored. The pathogenetic mechanism and the prognosis of injury are often determined based on the combination of patient history, physical examination, and ancillary tests. For example, occipital landing cases are often associated with a contralateral (bifrontal) brain contusion in addition to the injury at that site. A review at ≥6 h post-injury will often reveal a positive presentation even if the early imaging is not conclusive. Primary injuries in TBI (scalp lacerations, skull fractures, brain contusions, axonal injuries, etc.) are treated with debridement and suturing and conservative or surgical treatment. The indications for antibiotic treatment must be strictly controlled according to the Administrative Measures for the Clinical Application of Antimicrobial Drugs.

Infection treatment aims to mitigate definite pathogenic microbial infections, while that of prophylactic infection treatment is to mitigate the high-risk infection factors. This review suggests that lncRNAs in TBI mediate inflammatory damage caused by central injury through several inflammatory pathways, specific mechanisms, and immune-related responses and that the activation of some cells promotes the release of inflammatory factors and chemokines and complement formation. Thus, the use of antibiotics must be limited in the absence of clear pathogenic infections. However, treatment with anti-inflammatory agents, sympathetic excitation inhibitors, and antioxidants and agents that alleviate cerebral edema and exert neuroprotective effects are recommended in cases of secondary brain injury characterized by inflammatory factor release, oxidative stress, calcium overload, impairment of mitochondrial function, ferroptosis, apoptosis, pyroptosis, and autophagy. The therapeutic effect of these agents is limited, which can be attributed to the complex pathological mechanism of TBI. Based on these findings on immune-related inflammation, the following interventions can be recommended: one, blocking granulocyte infiltration into the brain; two, inhibiting and neutralizing pro-inflammatory and cytokine activity; three, inhibiting microglial activation and ferroptosis; four, blocking pattern recognition receptor activation of the inflammatory complex; five, inhibiting intrinsic and adaptive immune responses; six, preventing trauma systemic reactions after stress. These interventions may improve the prognosis of patients with TBI (Balu, 2014; Viet et al., 2021). Generally, the spleen and the brain are not correlated post-trauma. However, some studies have demonstrated that splenectomy decreases the number of B and T cells responding to TBI, which decreases lesion size and improves functional outcomes (Nizamutdinov and Shapiro, 2017). Blocking B and T-cell expansion and activation in the spleen exerted neuroprotective effects in other TBI models (Tobin et al., 2014). Thus, the therapeutic targeting of peripheral immune cells after TBI may enable the development of novel drugs and therapeutic modalities for secondary brain injury. We summarize the basic information, targets, pathways, etc. targets, pathways, etc. of lncRNAs related to traumatic brain injury (Table 1).

**TABLE 1:** The characteristics and targets point of traumatic brain injury (TBI)-related long non-coding RNAs (lncRNAs).

Name	Molecular size	Ensembl ID	Gene location	Target Point	Pathway	References
MALAT1	8,700 nt	ENSTG378938	11q13.1	miR-199b	IKKβ/NF-κB	Zhang et al., 2017b
NEAT1	4,000 bp	ENSTG283131	11q13	miR-365	Bax and Akt	Klec et al., 2019
Gm4419	1,730 bp	ENSMUST0000 0180671	Chr12: 21417911-21419803	P50/p61, mir-4661	NF-κB/ NLRP3	Yi et al., 2017
GAS5	4,983 bp	ENSG60674	1q25.1	miR-21 miR-124 p50, p21	mTOR	Mayama et al., 2016
MEG3	1,700 nt	ENSG55384	14q32.3	miR-548d-3p, miR-7a-5p	JAK-STAT, NLRP3	Mondal et al., 2015
CRNDE	1,059 bp	ENSTG 643911	16q12.2	-	-	Gao et al., 2017
HOTAIR	2,148 nt	ENSTG 100124700	12q13.13	ki-67	NRDP1/MYD88	Rinn et al., 2007
ZFAS1	2,653 nt	ENSG441951	20q13.13	-	Bax/caspase-3	Dong et al., 2018
NKILA	2,570 nt	ENSG105416157	20q13	miR-195	NF-κB	Yu et al., 2018
KCNQ1OT1	91,671 nt	ENSTG10984	11p15.5	miR-873-5p, TRAF6	TLR4, NF-K b	Zhang K. et al., 2021
COX2(Ptgs2)	755 bp	SGD:S000007281	MT:73758-74513	NLRP3/p65	NF-K b	Xue et al., 2019

*LncRNA, long non-coding RNA; NF-κB, nuclear factor kappa-light-chain-enhancer of activated B cells; NLRP3, Nucleotide-binding oligomerization domain, Leucine-Rich repeat and pyrin domain-containing 3; Bax, Bcl-2 Associated X protein; MCP-1, Monocyte chemoattractant protein-1; TNF-α, Tumor Necrosis Factor-α; IL-1β, Interleukin-1; Bcl-2, B cell lymphoma/lewkmia-2; CyclinD1, G1/S-specific cyclin-D1; IL-12, Interleukin-12; GSH, Glutathione; ROS, reactive oxygen species; mTOR, mammalian target of rapamycin; GFAP, glial fibrillary acidic protein; BrdU, deoxidation uracil nucleotides bromide; NGF, Nerve growth factor; AQP4, Recombinant Aquaporin 4; BBBB, Blood-brain barrier; Akt, Protein kinase B; JAK-STAT, Janus kinase/signal transducer and activator of transcription; IKKβ, an inhibitor of nuclear factor kappa-B kinaseβ; NRDP1, Neurotonin receptor degrading protein 1;MYD88, myeloid differentiation factor 88; Caspase, cysteinyl aspartate specific proteinase; TLR4, Toll-like receptor 4; TRAF6, TNF receptor-associated factor 6.*

Natalizumab, which inhibits the lymphocyte α4 integrin D signaling and consequently inhibits T-cell passage through the BBB, protects the brain against T-cell-mediated injury (Miller et al., 2003; von Andrian and Engelhardt, 2003; Polman et al., 2006). In patients with seizure onset after TBI, the administration of monoclonal antibody prevented and reduced seizure onset (Fabene et al., 2008). Thus, the reduction of immune cells and neutralization of intracerebral anti-brain antigen antibodies is a promising treatment for alleviating immune-mediated injury in patients with TBI. In secondary brain injury, lncRNAs mediate the onset and development of the immune responses (Table 2). TBI is associated with differential expression of lncRNA at the sites of brain injury. Hence, modulating lncRNA expression at the molecular level can potentially mitigate the inflammation injury-mediated damage in secondary brain injury. This can be a novel therapeutic strategy for clinicians to improve the prognosis of patients with TBI and decrease the burden for their families and society. Treatment at the genetic level is well-studied in oncology. However, genetic level treatment of extracerebral areas is a novel paradigm and it is a new challenge for the development of targeted drugs. The emergence of novel technologies will enable the elucidation of mechanisms underlying secondary brain injury and the development of novel treatment of TBI.

**TABLE 2:** Related long non-coding RNA (lncRNA) immune response in traumatic brain injury (TBI).

Name	Regulatory role (Function)	Period of immune response	Biological effect	References source
lncRNA-DC	Regulates dendritic cell differentiation and the binding of dendritic cell-stimulated activated T-cells to STAT3 and SHP1	Inherent immune response	Upregulation: neuroprotection Downregulation: pro-apoptosis	Wang et al., 2014
lncRNA MAF-4	Suppresses MAF expression and promotes the Th1 cell phenotype	Acquired immune response	Neuroprotection	Ranzani et al., 2015
NK cell	Inhibition of Th17 cell activation and expansion of NK cells in the brain using IL-2/anti-IL-2 monoclonal antibody complexes	Inherent immune response	Neuroprotection	Hao et al., 2011
lncRNA MALAT1	Mediates exosome production and inhibits IL-6	Inherent immune response	Upregulation: neuroprotection Downregulation: pro-apoptosis	Yang et al., 2017
lncRNA HOTAIR	Regulates granulocyte maturation and the expression of MYD88 and PCSK9	Inherent immune response	Upregulation: neuroprotection Downregulation: pro-apoptosis	Zhang et al., 2017a
lncRNA NEAT1	Regulates si-NEAT1 expression and promotes cytokine and chemokine release	Inherent immune response	Upregulation: neuroprotection Downregulation: pro-apoptosis	Imamura et al., 2014; Zhang F. et al., 2016
lncRNA GAS5	Regulates GRE and GR expression and ROS production	Inherent immune response	Upregulation: proapoptotic Downregulation: neuroprotection	Pickard et al., 2013
lncRNA COX2	Regulates the MYD88 and NF-κB signaling pathways and mediates the release of inflammatory factors	Inherent immune response	Upregulation: pro-apoptotic Downregulation: neuroprotection	Carpenter et al., 2013

*lncRNA, long non-coding RNA; NK, natural killer; ROS, reactive oxygen species; Th1, T helper 1 cell.*

## Summary and Future Perspectives

Long non-coding RNAs are a class of RNA with a length of >200 nt that is transcribed by RNA polymerase II. Additionally, lncRNAs exhibit a conserved secondary structure and mostly do not encode proteins. Furthermore, lncRNAs are involved in various biological processes, including signal transduction, immune response, inflammatory pathways, ion channel regulation, and cell cycle. Previous studies have reported that lncRNAs are differentially expressed in the brain tissue after TBI and that they mediate the pathophysiological process of inflammation in TBI. Inflammation is a critical pathological process in TBI. lncRNAs are associated with the activation, differentiation, and dysregulation of immune cells. Additionally, lncRNAs modulate T cells, B cells, and NK cells in autoimmune diseases and regulate the immune responses in TBI. The pathological mechanisms of secondary brain injury are complex and diverse. Secondary brain injury promotes systemic changes and traumatic stress in multiple organs. Hence, the therapeutic strategies for TBI must consider the functional impairments of other organs that accompany TBI (e.g., neurogenic pulmonary edema and stress ulcers). However, the mechanisms underlying differential lncRNA expression in TBI and lncRNA-mediated immune response must be elucidated in future studies, along with the correlation between different lncRNAs mediating inflammatory injury in TBI. Moreover, TBI is a major public health concern worldwide and is associated with poor survival rates and prognosis and societal and economic burden. The complex and diverse pathological mechanisms of TBI vary depending on the degrees of brain injury and may involve multiple organs and systems. Thus, the efficacy of traditional, conservative, and surgical treatments is poor. The mechanism of TBI must be elucidated to enable the development of novel therapeutic approaches. In conclusion, this review summarized the role of TBI-associated lncRNAs involved in inflammatory and immune responses that mediate the pathological process of secondary brain injury. The neuroinflammatory and immune response-mediated damages after TBI can be mitigated by regulating the expression of lncRNAs, which can be a potential novel treatment modality for patients with TBI. With the continued in-depth study of lncRNAs, specific lncRNAs are expected not only to be helpful in elucidating disease pathophysiological processes but also recent insights have shed light on the great potential of lncRNA as a non-invasive biomarker. We believe that these TBI-associated lncRNAs will provide a novel perspective for the development of therapeutic strategies for TBI.

## Author Contributions

J-PW wrote the manuscript and drew figures and tables. W-CD helped modify the tables. CL provided important guidance in the writing and revision of the manuscript. G-LX, QC, and GP provided overall supervision and edited the manuscript. RC and QC read and critically revised the manuscript. All authors contributed to the article and approved the submitted version.

## Conflict of Interest

The authors declare that the research was conducted in the absence of any commercial or financial relationships that could be construed as a potential conflict of interest.

## Publisher’s Note

All claims expressed in this article are solely those of the authors and do not necessarily represent those of their affiliated organizations, or those of the publisher, the editors and the reviewers. Any product that may be evaluated in this article, or claim that may be made by its manufacturer, is not guaranteed or endorsed by the publisher.

## Footnotes

1
http://www.exorbase.org
